# Rodents’ episodic-like memory: concepts, ageing, neurodegeneration, future

**DOI:** 10.1515/tnsci-2025-0392

**Published:** 2026-05-04

**Authors:** Andressa Gabriela Soliani, Beatriz Gangale Muratori, Jessica Santos Baptista, Suzete Maria Cerutti

**Affiliations:** Cellular and Behavioural NeuroPharmacology Laboratory, Department of Biological Sciences, Universidade Federal de São Paulo, Diadema, SP, Brazil; Department of Biological Science, Institute of Environmental, Chemical and Pharmaceutical Sciences, Universidade Federal de São Paulo, Diadema, SP, Brazil

**Keywords:** ageing, hippocampus, cholinergic system, novelty, memory consolidation, neurodegeneration

## Abstract

Episodic memory (EM), traditionally defined as the conscious recollection of *what, where*, and *when* events occurred, was long considered uniquely human due to its reliance on autonoetic awareness. However, behavioural criteria allow memory assessment based on observable features, enabling the study of EM-related processes in non-human species and leading to the concept of episodic-like memory (ELM) in animal models. We review conceptual advances and methodological challenges in ELM research, focusing on rodent models that provide mechanistic insights into neural circuits and molecular pathways supporting memory formation and persistence. Converging evidence highlights the role of hippocampal–prefrontal networks in integrating spatial, temporal, and emotional dimensions of experience. We also discuss data from our laboratory, which provides evidence for cellular and synaptic mechanisms, such as tagging-and-capture, ensemble allocation, and reactivation, that contribute to memory linking and consolidation in the fear-based ELM paradigm. These findings indicate that novelty-dependent recruitment of neuronal ensembles in the dorsal CA1 is impaired during ageing, leading to deficits in the persistence of weak experiences. Additionally, studies using cholinergic hypofunction models, a hallmark of late-onset Alzheimer’s disease (LOAD), reveal severe impairments in object recognition, spatial location, and integrated ELM, partially reversed by pharmacological interventions. Collectively, these data emphasise the translational relevance of ELM paradigms for modelling age-related memory decline and neurodegeneration. We also address open questions regarding neuromodulatory influences, sex differences, and the boundary between normal ageing and LOAD. By integrating conceptual, behavioural, and neurobiological perspectives, ELM approaches provide powerful tools for probing memory dynamics and informing therapeutic strategies.

## Introduction

### Conceptual framework: episodic memory

The earliest attempts to classify memory focused primarily on the duration for which information is retained within specific neural circuits. William James initially distinguished between primary and secondary memory, later reformulated as short-term (STM) and long-term memory (LTM) [1]. However, this time-based framework proved insufficient to capture the complexity of the mnemonic processes, prompting classifications grounded in the nature and content of stored information. Drawing on neurocognitive findings, Tulving [2] proposed a tripartite classification of LTM comprising semantic, episodic, and procedural. Within this context, Episodic Memory (EM) refers to the conscious recollection of personally experienced events situated within specific temporal and spatial contexts [2]. This feature distinguishes EM from semantic memory, which includes general knowledge, facts, and concepts that are independent of the circumstances in which they were acquired [3]. Episodic and semantic memory are therefore considered forms of declarative LTM and were long considered as uniquely human. In contrast, procedural memory supports the acquisition of skills and habits through experience and operates largely outside conscious awareness, characterising the non-declarative domain [1], 3]. EM relies on neural mechanisms that support the encoding, storage, and conscious retrieval of **What** happened, **Where**, and **When**, an autobiographical process that Tulving termed *autonoesis,* or mental time travel [4].

For decades, EM was considered exclusive to humans due to its dependence on autonoetic consciousness. However, the question whether non-human and non-verbal species can form EM has advanced substantially through studies distinguishing two complementary dimensions: (1) **The phenomenological dimension** refers to the subjective capacity to mentally relive past events or to project oneself through time, integrating what–where–when features of an episode and potentially incorporating its emotional or motivational significance. (2) **The behavioural dimension** evaluates EM through measurable performance, including discrimination of what occurred, where, and when; the integration of these components within neural circuits; and retrieval flexibility, defined as the ability to use stored information in novel or modified contexts. Together, these behavioural criteria allow researchers to operationalise and characterise EM in species that cannot provide verbal reports, distinguishing it from simpler associative processes and strengthening its relevance for comparative and translational neuroscience. In such cases, this capacity is referred to as Episodic-Like Memory (ELM). The integrative processes, linking event identity, spatial context, and temporal information, facilitate flexible retrieval and support adaptive behavioural responses [5], [6], [7], [8].

Pioneering studies by Clayton and Dickinson [6] provided the first evidence of ELM in birds. Subsequent research [9], [10], [11], [12], [13], [14] reinforced the view that non-human animals can recall information about events, their spatial context (where), and their temporal placement (what/when), even in the absence of demonstrable conscious awareness. An important aspect of the behavioural ELM characterisation concerns the temporal or “when” component. Although this dimension has previously received less attention in rodent studies, several behavioural tasks allow its assessment [15], 16]. These include paradigms in which animals learn temporal associations or sequences, as well as novelty-based tasks, variations of the spontaneous object recognition (SOR). These tasks exploit the innate preference of rodents to investigate the older object rather than the more recent one, enabling assessment of recency discrimination [17].

In contrast to tests that assess familiarity without incorporating contextual or temporal information, such as classical object recognition (OR), place recognition (PR) or context recognition (CR), which primarily evaluate where–which memory, the object–place–context recognition task (OPCRT) assesses what-where-when/which memory. Like OR, PR, and CR, the OPCRT relies on spontaneous exploration; however, it specifically requires the integrated processing of object identity, spatial location, and environmental context, all without emotional or motivational relevance [14], 18], 19]. These tasks depend on partially dissociable neural substrates, as will be described later. Collectively, behavioural, anatomical and neurochemical evidence demonstrates that rodents can retrieve not only the spatial and content-related components of an episode but also its temporal organisation [14], 18], [20], [21], [22], [23]. Converging with behavioural findings, neurochemical and anatomical data indicate that the prefrontal cortex and the hippocampal–entorhinal system contain time-sensitive representational mechanisms that integrate temporal information with spatial and contextual cues, supporting unified episodic-like representations [15], 22], 24].

In a comprehensive review, Chao et al. [25] emphasise that ELM, as originally conceptualised by Kart-Teke et al. [13] reflects more than the simple sum of its “what,” “where,” and “when” components. Instead, ELM emerges from the coordinated interaction of neural systems that jointly support the integration of object, spatial, and temporal information within a broader network architecture. Although the what, where, and when dimensions engage partially distinct neural substrates, it is their combined recruitment that gives rise to a distributed and interconnected engram representation [26]. Importantly, it is necessary to distinguish between the spatiotemporal features of the episode, such as the physical location and timing of the event, and the spatiotemporal dynamics of the neural circuits, including the coordinated activity of cell assemblies, that encode, integrate and consolidate these features into memory. The former refers to the experiential attributes of the event, whereas the latter reflects the underlying neural processes through which these experiential features are transformed into a coherent memory representation, or memory engram [27].

Silva and colleagues [12] provided an important contribution to the characterisation of ELM in rodents, particularly in the context of aversive experiences, by employing the Plus-Maze Discriminative Avoidance Task (PM-DAT) and examining the underlying neural circuits. This paradigm allows the dissociation of distinct behavioural components relevant to episodic-like processing. The “where” component depends largely on the recognition of a previously travelled route, which is guided primarily by proximal spatial cues supported by distal landmarks. In contrast, the “what” component corresponds to the aversive event itself, that is, the emotionally negative stimulus presented in one of the closed arms. Successful performance requires discriminating between the two enclosed arms, the aversive cue-associated arm and the neutral one, by integrating spatial information with the emotional significance of the experience. This demonstrates that rodents can combine spatial cues and emotional valence to contextualise an event, constituting a form of ELM. Importantly, the reliance on proximal cues observed in the PM-DAT highlights a feature that should also be emphasised in non-aversive paradigms designed to assess ELM. Many experimental models implicitly depend on proximal spatial information to support the “where” component, yet this dependence is not always explicitly depicted. Recognising the central role of proximal cues is essential for accurately interpreting memory performance and for distinguishing contextual, spatial and emotional contributions within episodic-like tasks.

Moreover, given its temporal stability and the specificity of its content, LTM is best understood as a set of distinct systems supported by partially dissociable neural networks. The characteristics of each system reflect both the specialisation of its underlying neural substrates and the particular representational demands it supports [5], 28]. It is now well established that external events are encoded in the specific cell engram as spatiotemporal patterns of neural activity. These patterns consist of precisely coordinated changes in neuronal and regional activation over time, which induce synaptic modifications and ultimately lead to the formation of an engram [29], [30], [31]. The formation of an ELM engram entails the convergence of multiple event-related dimensions. Clarifying how this integration is achieved at the cellular and systems levels will require a detailed characterisation of the neural mechanisms that encode, consolidate, and link these dimensions into a coherent memory trace.

### Neural circuitry and episodic-like memory: spatial and temporal scales

A growing body of evidence has substantially advanced our understanding of the neural architecture underlying ELM in both humans and animal models. Distinct subregions within the hippocampal formation (HF), notably CA1, CA3, and the dentate gyrus (DG) [5], [11], [12], [13], [14, [31], [32], [33], as well as the prefrontal cortex (PFC), play key roles in the encoding, integration, and consolidation of episodic information [34]. These regions operate within an integrated and distributed network responsible for representing the spatial, temporal, and emotional dimensions of events.

The HF primarily processes the spatial and temporal features of experience, supporting the encoding of detailed “what–where–when” information. Both the lateral (LEC) and medial (MEC) subdivisions of the entorhinal cortex (EC) maintain reciprocal connections with the DG and CA1/CA3 subfields, enabling the integration of object, spatial, and temporal features within a coherent episodic structure [24], 35], 36]. Importantly, the EC exhibits a highly modular and laminar organisation, characterised by parallel and selectively interconnected pathways that allow efficient routing and transformation of information across cortical–hippocampal networks [36]. LEC conveys multisensory and object-related information from the external environment, originating primarily from the perirhinal cortex (PRC), which is reciprocally interconnected with the PFC. PRC contributes strongly to novelty and familiarity discrimination, whereas PRC–PFC interactions support recency (temporal order) and object-in-place discrimination. In parallel, MEC receives strong input from the postrhinal cortex (POR), the rodent homologue of the human parahippocampal cortex, forming a major pathway for spatial and contextual information into the HF. MEC transforms these inputs into precise spatial codes through grid, non-grid, head-direction, and speed cells. Together, the POR–MEC–HF pathway supports the spatial and contextual (“where”) components of ELM, complementing the PRC–LEC pathway that conveys object-related (“what”) information and contributes to short-to intermediate–time-scale representations essential for recency and temporal order (“when”) [15], 16], 22], 35]. Consistent with this organisation, the LEC is recruited during the acquisition of ELM, supporting the binding of object, contextual, and temporal cues, and learning-tagged LEC neurons are also required for their recall, underscoring its associative role in both encoding and retrieval [33].

Connectivity between CA3 and the PFC is also crucial for integrating episodic elements [26], 37]. Consistently, ELM deficits have been associated with altered connectivity between the medial prefrontal cortex (mPFC), hippocampal subfields (CA1, CA3), and LEC [25], 33]. Given the HF’s central role in contextual and temporal encoding and in spatial navigation, it is likely that coordinated activity among hippocampal cell assemblies contributes to contextual representation and pattern separation across the broader ELM network. Connections among HF structures are also required for optimal performance in OPC/OPCRT tasks, which demand discrimination of objects, locations, and the integration of these features [14], 19], 33]. The PFC plays a central role in retrieving context-relevant memories and guiding goal-directed behaviour. Together, hippocampal and prefrontal circuits support the flexibility and specificity required for ELM, enabling adaptive use of information in situations requiring fine-grained discrimination [24], 32], 38]. In contrast, the amygdaloid complex (AC), particularly the basolateral amygdala (BLA), assigns *emotional valence* to events, encoding whether an experience is aversive or rewarding and regulating the strength and persistence of the memory accordingly [8], 9].

Building on earlier findings, our group investigated how transient suppression of dorsal CA1 activity affects discriminative avoidance memory by locally infusing muscimol, a potent GABAA receptor agonist, widely used to induce rapid and reversible neuronal inactivation. Muscimol was administered before either the acquisition or retrieval phases of the PM-DAT task, enabling us to dissociate the specific contribution of CA1 activity across distinct memory stages. In parallel, we observed an upregulation of CAMP-responsive element binding protein 1 (CREB-1) protein expression within hippocampal and prefrontal circuits, providing molecular correlates that support the functional impact of CA1 inactivation [39]. These findings confirmed the involvement of hippocampal–prefrontal networks in the formation of aversive ELMs. Furthermore, normative middle-aged rats exhibited impairments in this memory, which were reversed by pharmacological intervention with standardized *extract of Ginkgo biloba* (EGb), an effect associated with improved PFC function [40]. Together, these results indicate that discriminative avoidance memory relies on multiple behavioural parameters, including contextual and spatial discrimination, exploratory motivation associated with the emotional valence of stimuli, and behavioural flexibility. These components require coordinated integration between the hippocampal formation, the amygdaloid complex, and the prefrontal cortex for both encoding and retrieval, thereby reinforcing its characterisation as a form of ELM.

Neural circuits within the HF and PFC are particularly vulnerable to ageing [41], [42], [43]. Importantly, the integrity of the HF is differentially affected by ageing and neurodegeneration. Distinct subregions exhibit selective vulnerability. CA3 shows age-related increases in firing irregularity and hyperactivity, whereas CA1 displays reduced synaptic plasticity, impaired temporal coding, and deficits in place field stability [29], 44]. Moreover, the DG undergoes pronounced declines in pattern separation and reduced adult neurogenesis, compromising the fine-grained discrimination of similar experiences [45], 46]. Crucially, EC, particularly layer II neurons of the LEC, is among the earliest and most selectively affected regions in both normative ageing and late-onset Alzheimer’s disease (LOAD) [47]. Early degeneration of EC layer II neurons shows a strong correspondence with initial deficits in temporal and object-related memory, reflecting the essential role of this region in routing “what–where–when” information to the hippocampus. Pronounced EC atrophy has been described in LOAD, accompanied by reduced expression of brain-derived neurotrophic factor (BDNF) in both the EC and hippocampus. Notably, BDNF modulates tau hyperphosphorylation and mitigates tau-related toxicity, linking its dysregulation to the progression of early tau pathology [48]. Loss of EC integrity disrupts the transfer of object, contextual, and temporal information to hippocampal circuits, fragmenting the integration processes required for ELM. Combined with CA1/CA3 synaptic dysfunction and impairments in the dentate gyrus for pattern separation and neurogenesis, LEC vulnerability emerges as a central driver of the progressive deterioration of ELM observed during ageing and neurodegenerative disease [15], 22].

Disruptions in these networks compromise the formation of ELM, underscoring the importance of investigating the behavioural and neurochemical mechanisms that support memory consolidation and persistence during normative ageing. Such knowledge also provides valuable insights into analogous alterations observed in animal models of neurodegeneration, thereby informing the development of targeted therapeutic strategies. As detailed later in item 3, our group has investigated the consequences of reduced vesicular acetylcholine transporter (VAChT) expression (VAChT knockdown) in young adult female mice [49] and in aged female mice (unpublished data). In addition to the behavioural deficits, aged VAChT KD^HOM^ mice exhibited altered hippocampal expression of proteins associated with AD, further supporting a key role for cholinergic modulation in maintaining hippocampal integrity. Together, these results highlight the translational significance of cholinergic dysfunction, given that comparable memory deficits are hallmarks of age-related cognitive decline and neurodegenerative conditions such as AD.

Despite substantial progress, the precise neural circuitry and neurochemical pathways supporting EM and ELM remain incompletely understood. In particular, the dynamic interactions between distributed brain regions and the molecular mechanisms that govern memory encoding, retrieval, and long-term persistence during ageing and in AD require further investigation [26], 42], 50]. This knowledge gap is especially critical given that ELM is consistently disrupted under these conditions, reflecting the heightened vulnerability of the entorhinal–hippocampal–prefrontal network to ageing-related decline and neurodegeneration.

### Encoding and durability of ELM

Several research groups, including ours, investigate the neurochemical and cellular mechanisms underlying ELM in animal models, with a particular focus on how normative ageing and neurodegeneration disrupt these processes. Our research focuses on the neural circuits and neurochemical substrates underlying the encoding and long-term maintenance of both aversive and non-aversive ELM, examining features such as spatial cues linked to emotional valence and object recognition and object-location memory [49], 51]. We further assess pharmacological strategies with EGb across distinct experimental contexts, including exposure to environmental novelty in young rats [51], treatments in aged rats [40], and combination with strong training protocols in female mice under conditions of cholinergic signalisation deficit [49], aimed at preserving or restoring memory function. Additionally, we investigate how behavioural mechanisms modulate memory persistence within the fear-based ELM paradigm [52].

We showed that STM can be consolidated into LTM when novel information stabilises a weak memory trace, both at the level of neuronal ensembles and behavioural performance, in young rats. The temporal association between events, in which a novel experience occurring within a critical time window of training stabilises a weak trace (a process known as *behavioural tagging*), can transform a memory normally restricted to STM into LTM. This phenomenon has been demonstrated in aversive conditioning [53], [54], [55] and object recognition memory [56]. We reported that this process is disrupted by ageing. This impairment results from a reduction in novelty-recruited neuronal ensembles, impairing engram formation in the dorsal CA1 subfield and preventing LTM consolidation [52] (see Section 2 for details). A distinctive contribution of our work is the demonstration that this age-related reduction in novelty-driven neuronal recruitment occurs despite preserved contextual fear conditioning (CFC) acquisition in normative ageing. This dissociation enabled us to distinguish ageing effects on acquisition from those on consolidation, providing compelling evidence that ageing selectively impairs novelty-driven modulatory processes essential for memory consolidation.

## Ageing disrupts episodic-like memory (ELM) consolidation by reducing tag-and-capture processes and impairing temporal–spatial linking

As highlighted before, EM or ELM are encoded and allocated to specific neuronal ensembles, which change synaptic connections in multiple regions, including the HF, AC, and prefrontal cortex, and the coordinated activity of these ensembles leads to memory retrieval. The nature of these changes is thought to underlie the duration of the memory trace. Following encoding, memories undergo consolidation at both the cellular/synaptic and systems levels, allowing them to persist in the brain [57]. This process is selective, meaning that some experiences are more likely to be stored in LTM than others [55].

Most EM are rapidly encoded but not consolidated, showing substantial trace decay over time. Why do some memories become long-lasting while others quickly vanish? In the young brain, novelty and/or other salient stimuli have been shown to be a critical factor for memory consolidation by activating brain circuits and molecular pathways that enhance consolidation at the cellular level through modulating protein synthesis, resulting in more stable memories [52], 55], 58].

The cellular basis of this phenomenon is explained by the synaptic tagging and capture (STC) hypothesis, originally proposed by Frey and Morris (1997), which remains widely recognised in the contemporary neuroscience literature [59]. Briefly, the STC hypothesis proposes that synapses activated by an experience are “tagged” and later capture plasticity-related products (PRPs; mRNA and proteins) produced in the same pool of neurons. In hippocampal slices, weak tetanic stimulation creates a tag and induces a transient form of synaptic potentiation, which can be converted to an enduring form of long-term potentiation if shortly preceded or followed by strong stimulation that is sufficiently strong to provide PRPs to both pathways [59]. In behavioural terms, weak memories can be transformed into LTM when PRPs are produced in response to novel or arousing stimuli, leading to memory consolidation. Several behavioural studies support this tag-and-capture mechanism, in which weak memories that would otherwise be forgotten are tagged and subsequently rescued by surrounding environmental novelty within a short temporal window [52], 55], 58].

An everyday example of this phenomenon occurs when attending a scientific conference and presenting your research. The next day, you can recall not only your presentation but also other seemingly trivial details that would have otherwise been forgotten, such as buying a coffee or what you ate for lunch. This incidental encoding is a critical feature of human episodic memory. Interestingly, recalling your presentation often facilitates the recall of other temporally related memories, demonstrating the richness and interconnectedness of episodic timelines.

There is compelling evidence that contextual memories encoded close in time are more likely to be co-allocated to an overlapping ensemble in the hippocampus [60]. This results in memories that are behaviourally linked, such that recalling one facilitates recalling the other. However, this cognitive ability changes across our lifespan. Aged mice exhibit memory-linking deficits, reduced neuronal excitability, and decreased ensemble overlap in the dorsal CA1 (dCA1) subfield [60]. Some studies have shown that tag-and-capture neural mechanisms and behavioural outcomes are impaired during ageing [61]. Although promising, these two lines of evidence regarding how ageing may affect the creation of ELM remain poorly underexplored.

To address this, we developed a weak contextual fear conditioning (wCFC) protocol, in which male rats learn to associate a shock with a specific context (A) (what-where association). In this protocol, one single weak footshock (0.3 mA) induces a memory that lasts approximately 30 min (STM) but is not consolidated. To assess the effect of novelty, 30 minutes later, animals were exposed to a new context without a shock (context B) in the same room (distal cues unchanged) and tested one day later [52]. We had two main hypotheses: i) If memories are co-allocated and linked at both cellular and behavioural levels, we would expect the creation of an integrated LTM representation. This would involve the integration of what (shock), when and where (temporal and spatial context, respectively) information. Therefore, animals should express LTM fear memory in both contexts A and B, although they never experienced a shock in the latter. Considering that normal ageing is associated with reduced hippocampal synaptic plasticity mechanisms, we predicted that aged rats (15–18 months old) would not result in LTM formation. As expected, in young animals, the procedure resulted in a long-lasting memory persisting for at least seven days, consistent with episodic memory’s defining feature: the storage and recall of events and their spatial and temporal relation [52]. Mechanistically, using fluorescent *in situ* hybridization (FISH) of immediate early gene expression, we found memory linking and consolidation depended on the magnitude of a shared ensemble in the dorsal CA1 (∼27 % overlap). Nevertheless, in aged animals, exposure to a new context following wCFC failed to induce memory consolidation. Significant changes were observed in the aged dCA1, including a reduction in “memory” neurons, indicating decreased neuronal allocation to engrams supporting new information, and a decrease in the number of reactivated neurons, with only approximately 15 % ensemble overlap [52].

Post-encoding reactivation of neuronal patterns present during the initial experience is crucial for consolidation, as optogenetically silencing ensemble-specific reactivation shortly after learning impairs consolidation [62]. Both successful tag-and-capture and memory linking depend on the reactivation of an overlapping ensemble [60], 61]. Thus, diminished ensembles’ reactivation during new context exposure is one major mechanism underlying the consolidation deficit in aged animals. Additional mechanisms contribute to the tag-and-capture phenomenon. In aged hippocampal slices, dCA1 synapses fail to exhibit STC properties, which is associated with a reduced pool of PRPs [63]. This reduction promotes synaptic competition rather than cooperation, impairing the consolidation and persistence.

Consistent with this view, in our study, we observed fewer Arc (activity-regulated cytoskeleton-associated protein) mRNA-positive neurons in the dCA1 subfield of aged rats. Arc is an immediate early gene essential for long-lasting synaptic plasticity and memory consolidation [52]. It is rapidly expressed following neuronal activation, and its mRNA is targeted to newly activated excitatory synapses for local translation [64]. This process links synaptic activity to protein synthesis-dependent synaptic plasticity, supporting Arc as a strong candidate for a PRP. Thus, the age-related reduction in Arc expression in dCA1 in response to novel context exposure may exacerbate synaptic competition, further inhibiting memory consolidation.

Evidence from the literature indicates that periencoding novelty/arousal stimuli within a defined temporal window can trigger the release of hippocampal neuromodulators – dopamine, norepinephrine, and acetylcholine – which in turn modulate the activity of the corresponding neuronal ensemble. Modulatory brain systems, such as the dopaminergic locus coeruleus-dCA1 pathway, are influenced by ageing [65]. The locus coeruleus (LC), a brainstem nucleus, is essential for memory linking by modulating neuron excitability in the hippocampus’s dCA1 area through dopamine signalling via D1 receptors [66]. This modulation facilitates the association of different memories and events encoded over time [66]. Additionally, this pathway is crucial for the tag-and-capture behavioural effect [58]. Interestingly, Arc protein expression is induced by D1 receptor activation, along with increased expression of pCREB and BDNF levels, all of which are involved in consolidation [67]. Moreover, CREB has been shown to increase neuronal excitability, and reduced pCREB levels are observed in the aged hippocampus and in the LC [68]. This suggests that the LC dopaminergic system is a critical regulator of synaptic and cellular events essential for enduring plasticity and memory.

Based on these considerations, a plausible mechanism emerges: ageing reduces LC-driven dopaminergic signalling, leading to reduced neuronal reactivation, lower Arc expression and a diminished PRPs pool. This, in turn, shifts synaptic interactions from cooperation to competition, ultimately disrupting the tag-and-capture processes required for memory consolidation and persistence. Notably, Arc plays a critical role in synaptic scaling through AMPA receptor endocytosis, inverse synaptic tagging, and the regulation of structural plasticity by modulating spine density and morphology. Therefore, its reduction in the aged dCA1 may further disrupt the balance between potentiation and depression, a crucial process for maintaining functional memory networks [69]. Future research should further investigate how ageing alters LC function and downstream hippocampal plasticity, with the goal of elucidating mechanisms underlying ELM consolidation deficits.

One critical aspect of ageing is the increased vulnerability to neurodegenerative diseases, with LOAD being the most prevalent. A hallmark symptom of LOAD is the impairment in the formation and persistence of ELM that rely on HF and PFC circuits, largely associated with the progressive loss of cholinergic function in these regions [70].

## Severity of cholinergic impairment determines episodic-like memory deficits in mice

The cholinergic system plays a pivotal role in memory formation by modulating neuronal and astrocytic morphology and function in rostral brain structures. In particular, cholinergic projections to the HF and PFC are essential for regulating synaptic plasticity, excitability, and cognitive flexibility, processes critical for episodic-like memory (ELM) [71]. Cholinergic hypofunction has been implicated in hippocampus-dependent memory deficits in patients with LOAD. In AD, degeneration of basal forebrain cholinergic neurons reduces cholinergic tone in the HF and cortex, contributing to impairments in synaptic plasticity and memory consolidation [72].

Our group has investigated the consequences of reduced vesicular acetylcholine transporter expression (VAChT knockdown) in young adult female mice [49] and in aged female mice (unpublished data). Our recent data showed that young mice (3–6 months old) with a 65–70 % reduction in VAChT expression (knockdown homozygotes, VAChT-KD^HOM^) exhibited cognitive deficits in distinct paradigms [49]. As discussed previously, the Object Recognition (OR) and Object Location (OL) tasks probe the “what” and “where” components of ELM, respectively [21]. In these tasks, wild-type (WT) mice preferentially explore the novel object or location during the probe session. In contrast, female VAChT-KD^HOM^ mice display marked impairments in these tasks, whereas mice with a moderate VAChT reduction (45 %, VAChT-knockdown heterozygotes, VAChT KD^HET^) do not show significant impairments. This suggests that a moderate reduction in VAChT levels is still sufficient to support learning during the familiarisation phase. Notably, all animals underwent two 15-min familiarisation sessions, a relatively strong training protocol, which enabled VAChT-KD^HET^ mice to successfully identify the novel object during testing. In the PM-DAT, which requires the integration of ELM components, WT mice learn to avoid the enclosed arm associated with aversive cues by binding its spatial location to emotional valence. Interestingly, both VAChT-KD^HOM^ and VAChT-KD^HET^ females successfully discriminated the aversive arm during the training session, indicating preserved short-term memory (STM) but failed to retain this information after 24 h. In the PM-DAT, which requires the integration of ELM components, WT mice learn to avoid the enclosed arm associated with aversive cues by binding its spatial location to emotional valence. Interestingly, both VAChT-KD^HOM^ and VAChT-KD^HET^ females successfully discriminated the aversive arm during the training session – indicating preserved STM but failed to retain this information after 24 h (LTM). This aligns with prior evidence showing that ELM deficits in mouse models often reflect impairments in retrieval rather than encoding [73]. In contrast, WT mice avoided the aversive arm both during training and at test. Collectively, these findings indicate that different degrees of cholinergic reduction disrupt spatial learning and long-term memory consolidation [49]. More recently, we found that aged VAChT-KD^HOM^ mice exhibit pronounced deficits in STM and LTM of aversive ELM, alongside impairments in OR and OL memory (manuscript under revision). These results parallel our observations in young mutants, although in the younger cohort, the impairment was restricted to LTM [49].

Ageing further intensifies the vulnerability associated with reduced cholinergic signalling. Aged VAChT-KD^HOM^ mice show substantially greater impairments in hippocampal-dependent memory than young mutants, indicating that age-related decline exacerbates the functional consequences of cholinergic hypofunction. This heightened susceptibility extends the phenotype beyond the deficits observed in young animals. This framework provides a plausible explanation for the selective LTM impairment seen in young VAChT-KD^HOM^ females: consolidation processes appear particularly sensitive to diminished cholinergic tone, and ageing amplifies this susceptibility. Elucidating how ageing interacts with cholinergic hypofunction to compromise memory circuits is therefore crucial for understanding the neurobiology of cognitive decline and for identifying therapeutic targets relevant to age-related disorders such as AD. ​​This age-related vulnerability further destabilises hippocampal circuitry, resulting in a broader and more severe phenotype than that seen in young mutants.

This framework offers a plausible explanation for why long-term, but not short-term, memory is selectively compromised in young VAChT KD^HOM^ females: consolidation processes seem particularly sensitive to cholinergic insufficiency, and ageing intensifies this failure. Understanding how ageing interacts with cholinergic hypofunction to compromise memory circuits is therefore essential, both for clarifying the neurobiology of cognitive decline and for identifying therapeutic targets relevant to age-related disorders such as AD. Moreover, pharmacological intervention with EGb improved performance in a dose-dependent manner, reversing deficits in both OR memory and PM-DAT during STM and LTM. Overall, these findings reinforce the relevance of examining ELM and its individual components, as this framework provides critical insights into the mechanisms underlying memory decline in AD. Such knowledge is essential for guiding future investigations aimed at developing therapeutic strategies or identifying novel targets capable of mitigating cholinergic- and age-related impairments.

## Future directions

Despite substantial advances in our understanding of EM and ELM, fundamental questions remain regarding the cellular and circuit-level mechanisms underlying encoding, consolidation, retrieval, and long-term persistence. The precise functions of hippocampal subregions, including the dorsal and ventral dentate gyrus, CA1, and CA3, and their interactions with the PFC, EC, and AC remain poorly defined. Moreover, different memory systems exhibit distinct vulnerabilities to interference, with EM particularly prone to distortion and forgetting during ageing or in AD [13], 49], 52], 70].

Animal models are indispensable for dissecting the mechanisms of EM and ELM, allowing precise anatomical, pharmacological, physiological, genetic, and molecular manipulations that are not feasible in humans. These models have been crucial for understanding the temporal dynamics of memory formation, as evidence indicates that ELM can emerge within 50–60 min and persist for at least 24 h, challenging the notion that EM should be conceptualised solely as an LTM system [9], 52], 74].

These observations raise pivotal questions: Which aspects of EM/ELM – *what, where,* or *when* – are most susceptible to decline? Are similar mechanisms responsible for the spatial memory and object recognition deficits observed in neurodegenerative models? How does ageing impact these processes, and to what extent can impairments be reversed? Importantly, do sex differences influence the formation and persistence of ELM, necessitating studies in both males and females? Modulatory systems, including the locus coeruleus and basal forebrain, are essential for ELM formation and stability, yet are particularly vulnerable to ageing, potentially compromising synaptic plasticity in downstream regions such as the hippocampus and prefrontal cortex [52], 65], 71], 75]. The contributions of neuromodulators – dopamine, serotonin, acetylcholine – and neurotrophic factors such as BDNF to memory persistence remain an active area of investigation.

Our group has contributed novel insights into these questions. We developed a paradigm to assess ELM in rodents using CFC combined with exposure to a novel context within a brief temporal window, enabling integrated LTM for *what-where-when.* Using this approach, we demonstrated that ageing impairs ELM, providing mechanistic insights into memory decline [52]. Furthermore, our findings show that young female mice with reduced VAChT expression exhibit impairments in OR and OL memory. In the PM-DAT, these animals display selectively disrupted LTM while preserving STM and also show alterations in anxiety-like behaviour [49]. However, when this cholinergic deficit is combined with ageing, the impact becomes markedly more severe: aged VAChT KD^HOM^ mice exhibit robust impairments in both STM and LTM in the PM-DAT, as well as pronounced LTM deficits in the OR and OL tasks. This pattern suggests that ageing amplifies the vulnerability of memory circuits to reduced cholinergic signalling, broadening the cognitive impairments beyond those observed in young mutants.

Taken together, these findings highlight the complex interplay between neural circuits, neuromodulatory systems, and ageing in shaping ELM. They underscore the need for integrative approaches that combine behavioural, molecular, and circuit-level analyses to better understand memory decline and identify potential therapeutic targets for ageing and neurodegenerative diseases. Finally, these findings indicate that ELM is not restricted to long-term processes and can, in fact, be assessed within STM timescales.
